# Severe cardiopulmonary complications of ketamine: Acute pulmonary Edema in a cardiac patient

**DOI:** 10.1093/omcr/omaf063

**Published:** 2025-08-20

**Authors:** Tahir Shahzad, Zain A Bhutta, Muhammad Abd Ur Rehman

**Affiliations:** Department of Emergency Medicine, Hamad Medical Corporation, Al Rayyan Road, Doha 3050, Qatar; Department of Emergency Medicine, Hamad Medical Corporation, Al Rayyan Road, Doha 3050, Qatar; Department of Emergency Medicine, Hamad Medical Corporation, Al Rayyan Road, Doha 3050, Qatar

**Keywords:** ketamine, procedural sedation, Acute pulmonary edema, cardiogenic shock

## Abstract

Ketamine use in the emergency department is more widespread than ever due to its favorable safety profile and combined analgesic and anesthetic properties. Its ability to stimulate the cardiovascular system via the sympathetic nervous system is particularly important, as patients can develop hemodynamic instability. However, concerns remain regarding the risk of decompensated cardiac function due to extreme tachycardia and severely elevated systemic blood pressure. In this case, a middle-aged male with a known history of coronary artery disease and congestive heart failure deteriorated significantly after ketamine was administered for sedation to facilitate a computed tomography pulmonary angiogram. He developed acute pulmonary edema and cardiogenic shock, requiring mechanical ventilation and inotropic support. Therefore, it is advisable to use ketamine with caution in patients with underlying heart failure. Further research is needed to establish a causal relationship between ketamine and cardiac decompensation leading to cardiogenic shock and pulmonary edema in selected patients.

## Introduction

Ketamine is a widely used medication in emergency departments (ED) worldwide for procedural sedation, rapid sequence intubation, and the management of refractory asthma, chronic obstructive pulmonary disease (COPD), and seizures. It offers dose-dependent anesthetic, analgesic, bronchodilatory, and antiemetic effects, with a strong safety profile due to its ability to preserve airway reflexes and its cardiovascular stimulatory properties [1]. Ketamine is considered effective and safe in managing acute asthma or COPD exacerbations due to its bronchodilatory and sedative effects, which help improve synchronization with non-invasive ventilation. For rapid sequence intubation in asthma patients, a dose of 1–2 mg/kg is typically used [2]. Ketamine is associated with certain side effects, including tachycardia, hypersalivation, elevated systemic arterial pressure, psychomimetic reactions, and emergence delirium. In this report, we describe a complex clinical case involving a patient who initially presented with features suggestive of an asthma or COPD exacerbation. Although the patient showed improvement with initial treatment, he suddenly developed acute pulmonary edema following ketamine administration for sedation. This case is notable due to the diagnostic challenge it posed in the ED, with uncertainty surrounding whether the pulmonary edema was caused by underlying heart failure (HF) or was drug-induced.

## Case report

Emergency Medical Services brought in a 58-year-old male with complaints of shortness of breath and central chest pain that began at his workplace in the afternoon. The pain was intermittent, radiating to the left shoulder, and was not accompanied by nausea, vomiting, diaphoresis, syncope, or fever. He was a known case of hypertension, type II diabetes mellitus, dyslipidemia, obstructive lung disease, congestive HF, and coronary artery disease (CAD). He had a history of percutaneous coronary intervention with the placement of two drug-eluting stents approximately six months prior to presentation, and his last documented ejection fraction (EF) was 30%. He was an ex-smoker with no active substance use and no significant family history.

**Figure 1:**
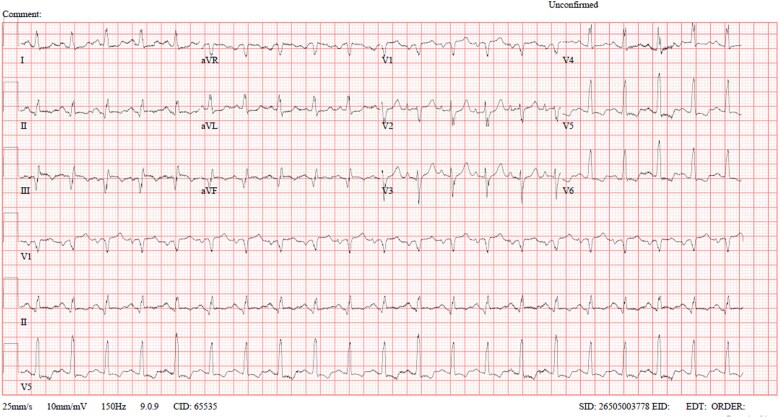
ECG showing sinus tachycardia with ST-elevation in inferior leads along with ST-depression & T-wave inversion in lateral leads.

Upon initial assessment, decreased air entry was noted in both hemithoraces, along with scattered bilateral rhonchi. He was not using accessory muscles of respiration and was able to speak in full sentences. Initial vital signs showed tachycardia (heart rate: 121 beats/min), mild tachypnea (respiratory rate: 24 breaths/min), and oxygen desaturation (SpO₂: 94% on room air). He was afebrile (temperature: 37°C) with a blood pressure of 152/64 mmHg. The electrocardiogram (ECG) revealed sinus tachycardia with ST-segment elevation in the inferior leads, along with ST depression and T-wave inversion in the lateral leads (Fig. 1). Aside from tachycardia, the remaining findings were consistent with his previous records (Supplementary Material).

**Figure 2:**
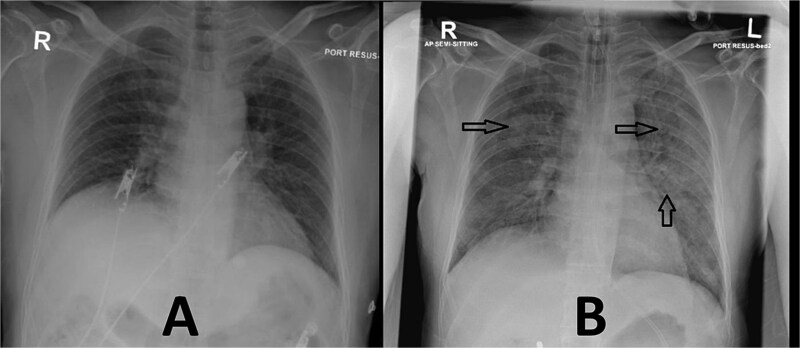
(A) Upon arrival to the emergency department. (B) After ketamine administration (arrows are pointing towards bilateral fluffy infiltration and vascular cephalization).

Point-of-care blood gas analysis revealed hypercapnia, so he was initially managed as a case of obstructive lung disease exacerbation with nebulized salbutamol, ipratropium, and systemic corticosteroids. Given his significant medical history and the presence of chest pain, cardiac enzymes were also sent. The chest X-ray showed no evidence of infection or fluid overload (Fig. 2A). Other laboratory investigations revealed a mildly elevated D-dimer (0.66 mg/l FEU) and two comparable sets of high-sensitivity troponin-T levels (20 and 23 ng/l) (Table 1).

**Table 1:** Laboratory findings (at the time of presentation).

Blood Investigations	Patient’s values	Normal Range
White blood cell (x 10^3^/μl)	14.7	4.0–10.0
C-reactive protein (mg/l)	4.4	0.0–5.0
D-dimer (mg/l FEU)	0.66	0.00–0.49
Creatinine (μmol/l)	81	62–106
Troponin T (ng/l)	20, 23^a^, 67^b^, 182^b^, 494^b^	3–15
COVID—19 Antigen	Negative	
pH	7.267	7.35–7.45
PCO2 (mmHg)	63	21–69
PO2 (mmHg)	21	25–40
HCO3 (mmol/l)	28.7	23–29

^a^3 h after the initial troponin.

^b^After administration of Ketamine.

After initial management, the patient showed clinical improvement, except for persistent tachycardia. Due to the ongoing tachycardia and elevated age-adjusted D-dimer, a computed tomography pulmonary angiography (CTPA) was requested. However, while in the CT scanner, the patient was unable to lie flat due to claustrophobia. Given his history of obstructive lung disease, 50 mg of intravenous ketamine was administered to facilitate the scan. A few minutes after ketamine administration, the patient became combative, followed by a decreased level of consciousness. Due to his extreme agitation, it was initially difficult to obtain an accurate oxygen saturation reading. He was placed on 15 L of oxygen via a non-rebreather mask, but his oxygen saturation remained persistently low, ranging between 70%–80% on the monitor. His end-tidal carbon dioxide levels fluctuated between 20 and 30 mmHg. His heart rate spiked to 146 beats per minute, followed by a drop in blood pressure to 89/63 mmHg, prompting the initiation of dobutamine and noradrenaline infusions. Non-invasive ventilation was used as a bridge for pre-oxygenation, and rapid sequence intubation was performed to secure the airway and improve oxygenation and ventilation. A repeat chest X-ray taken two hours after ketamine administration revealed extensive pulmonary edema (Fig. 2B).

Serial measurements of high-sensitivity troponin T were performed following the episode, revealing a progressive elevation after ketamine administration (Table 1).

**Figure 3:**
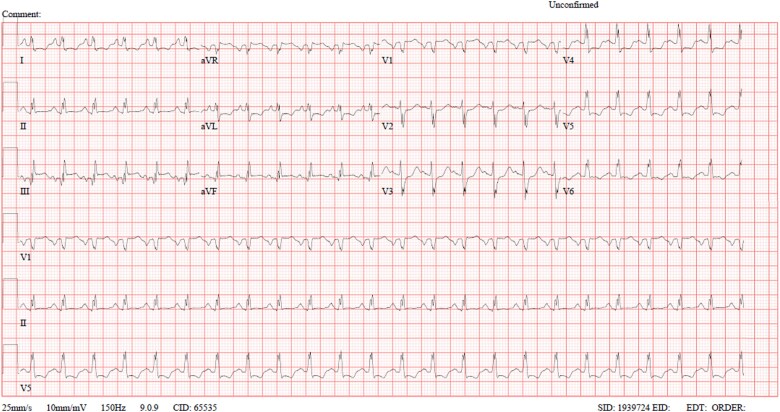
ECG done after pulmonary edema episode.

A repeat ECG showed persistent ST-segment elevation in the inferior leads and ST-segment depression in the lateral leads, with no evidence of progressive ischemic changes (Fig. 3).

The CTPA showed no evidence of pulmonary embolism, aortic dissection, lung parenchymal abnormalities, or pleural effusion. An echocardiogram revealed a critically reduced EF of 26%. Based on the repeat chest X-ray findings and the rising trend of high-sensitivity troponin, the patient was admitted to the Cardiac Care Unit with a diagnosis of ketamine-induced cardiogenic shock and myocardial injury.

**Figure 4:**
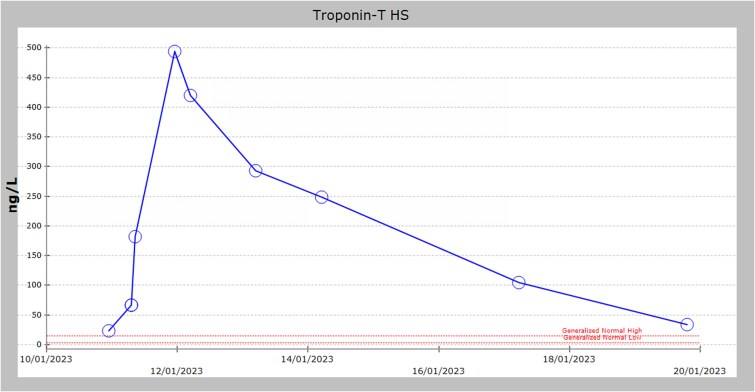
High-sensitivity troponin T.

A coronary angiogram was performed, revealing patent previously placed stents with no evidence of new obstruction (Supplementary Figure 2). By the following day, his troponin T levels began to decline (Fig. 4). He was extubated on day 3 and subsequently discharged on day 7 of hospitalization with optimized HF medications.

## Discussion

Cardiogenic shock and myocardial injury associated with ketamine use are rarely documented and are not recognized as established adverse effects in either adults or the pediatric population [3–6]. Ketamine exerts central sympathetic stimulation and inhibits neuronal catecholamine reuptake, leading to cardiac stimulation and elevated myocardial oxygen demand [7]. The majority of reported cases have come from anesthesiology settings involving patients undergoing elective procedures [8, 9]. Excessive sympathetic stimulation can overwhelm the cardiovascular compensatory mechanisms, potentially leading to pulmonary edema, as previously reported in cases involving the combined use of ketamine and cocaine [10]. In this case, the patient’s history of significant HF and CAD likely predisposed him to acute decompensation and cardiogenic shock, with ketamine-induced sympathetic stimulation further compromising an already weakened cardiac function. To the best of our knowledge, only one case has been reported from an ED in which ketamine administration resulted in acute pulmonary edema in a patient with underlying CAD [5]. The coronary angiogram in our case further supports this diagnosis, showing no evidence of new coronary occlusion.

**Figure 5:**
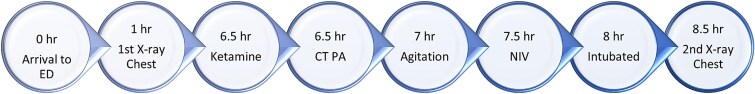
Timeline.

Ketamine is commonly used to manage asthma or COPD exacerbations due to its bronchodilatory and anti-inflammatory properties. Its sedative effects also aid in optimizing non-invasive ventilation in agitated or restless patients experiencing acute exacerbations [11]. Based on these benefits, ketamine was administered to our patient, given his known history of obstructive pulmonary disease. However, despite its favorable profile, the presence of underlying cardiovascular disease and an unexpected trigger—claustrophobia—led to serious complications, including cardiogenic shock and pulmonary edema. We hypothesize that the patient already had elevated sympathetic activity due to claustrophobia, which was further intensified by ketamine-induced sympathetic stimulation. Given his baseline HF and reduced EF, his cardiovascular system was unable to compensate for the sudden surge in sympathetic output, resulting in acute pulmonary edema. Another diagnostic challenge was differentiating between ketamine-induced psychosis, a known side effect, and hypoxia-driven behavioral changes [12]. Ketamine-induced agitation and restlessness can closely mimic the signs of acute hypoxia, contributing to a significant delay in recognizing and managing the hypoxia, as illustrated in the patient’s timeline (Fig. 5).

Recent studies indicate that etomidate may serve as a suitable alternative; however, there is no clear consensus favoring its use over ketamine. Much of the existing research has concentrated on the risks associated with intubation. In the context of tracheal intubation, etomidate has been associated with a reduced risk of post-induction hypotension [13] and greater hemodynamic stability [14] compared to ketamine. A recent meta-analysis of 32 randomized controlled trials examining procedural sedation and analgesia in emergency settings reported that ketamine had the lowest rates of respiratory adverse events, including apnea and hypoxia, whereas etomidate was associated with the lowest incidence of hypotension [15].

In conclusion, the patient developed cardiogenic shock and acute pulmonary edema likely due to ketamine-induced sympathetic stimulation overwhelming the heart's compensatory mechanisms. This case highlights the importance of using ketamine with caution in individuals with significant cardiac history and being vigilant for potential complications to enable timely intervention. It is also crucial to differentiate between hypoxia-induced behavioral changes and ketamine-related psychomimetic effects. Further clinical research and trials are needed to better understand and confirm the causal relationship between ketamine and cardiogenic shock or pulmonary edema.

## Supplementary Material

Supplementary_Materials_omaf063
