# Supplementary material for: Severe cardiopulmonary complications of ketamine: Acute pulmonary Edema in a cardiac patient
Source: Oxf Med Case Reports. 2025 Aug 20;2025(8):omaf063. doi: 10.1093/omcr/omaf063 (PMC12365973; doi:10.1093/omcr/omaf063)
Supplement: Supplementary_Materials_omaf063 [file supplementary_materials_omaf063.docx]

**Severe Cardiopulmonary Complications of Ketamine: Acute Pulmonary Edema in a Cardiac Patient**

**Supplementary materials:**


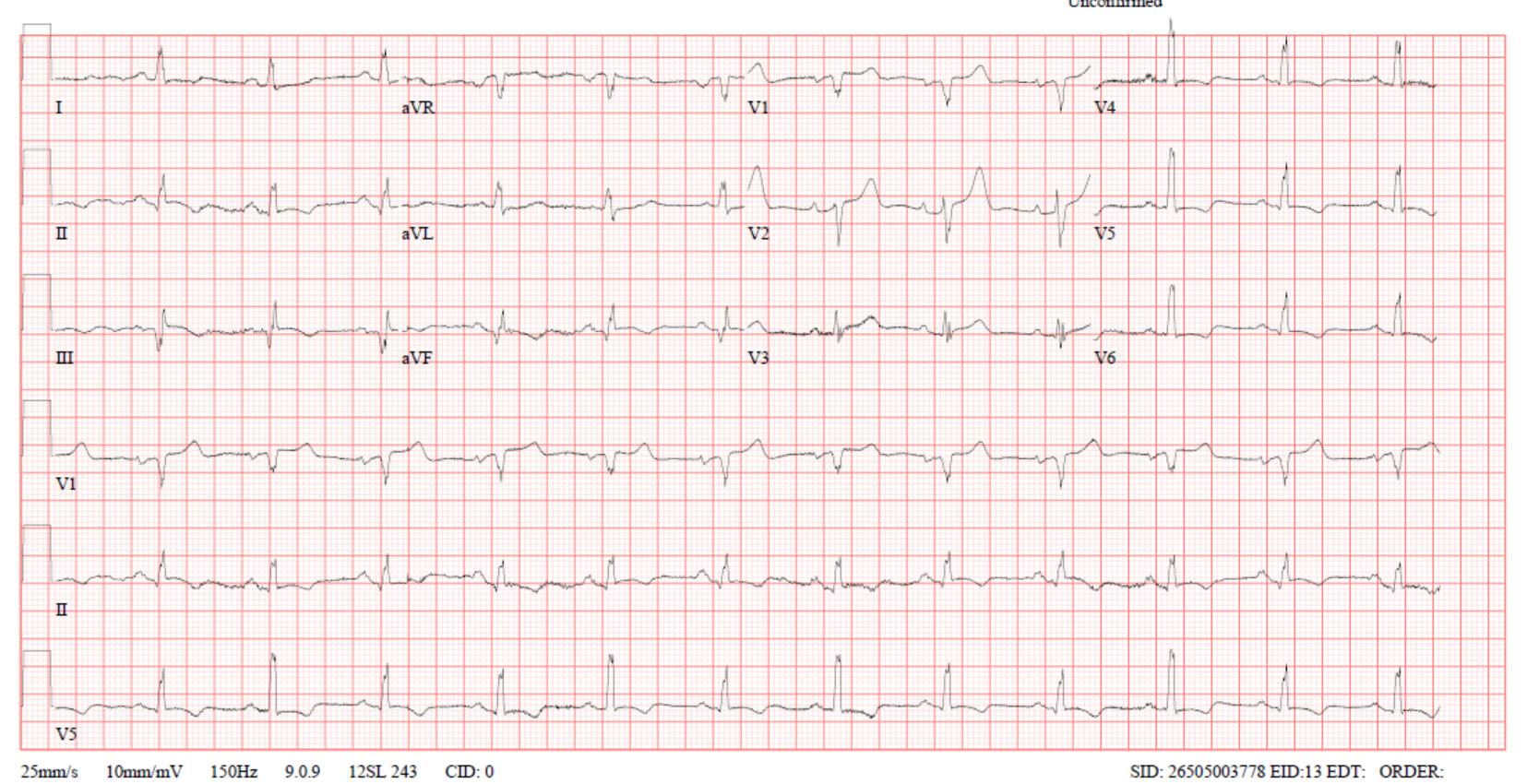


Figure 1: Baseline ECG of the patient, retrieved from his previous electronic medical records, shows ST elevation in the inferior leads along with ST depression and T-wave inversion in the lateral leads.


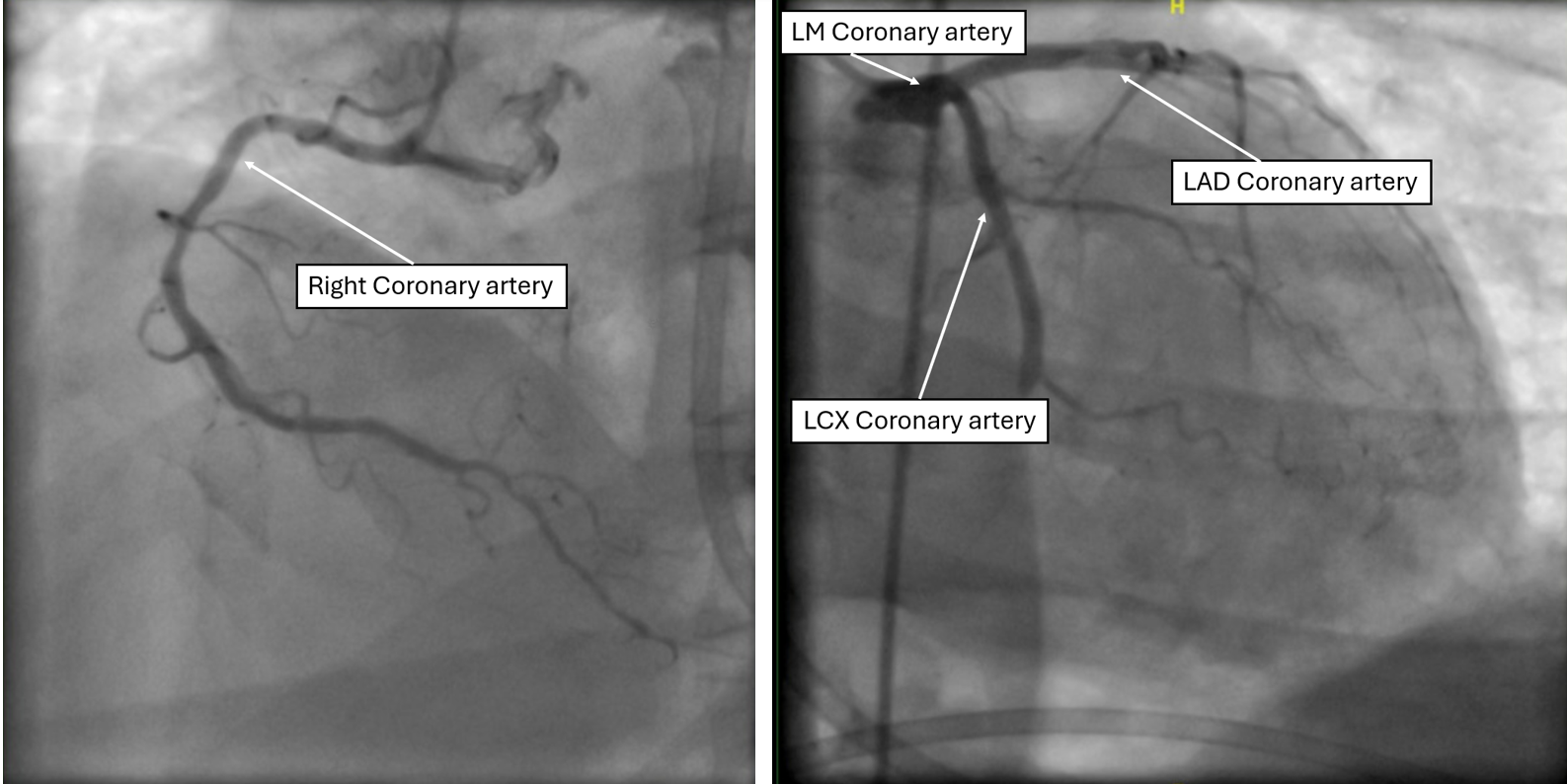


Figure 2: Coronary angiography of the patient showing patency of all major coronary vessels. LM: Left Main, LAD: Left Anterior Descending, LCX: Left Circumflex.
